# Rethinking exercise targets: sex differences in the road to a longer life

**DOI:** 10.1093/ndt/gfag053

**Published:** 2026-03-10

**Authors:** Amaryllis H Van Craenenbroeck, Olga Balafa, Magdalena Jankowska, Nilufar Mohebbi, Ana Garcia Prieto, Lucia Del Vecchio

**Affiliations:** Department of Nephrology, University Hospitals Leuven, Leuven, Belgium & Department of Microbiology, Immunology and Transplantation, Nephrology and Renal Transplantation Research Group, KU Leuven, Leuven, Belgium; Department of Nephrology, General Hospital of Ioannina “G. Hatzikosta”, Ioannina, Greece; Department of Nephrology, Transplantology and Internal Medicine, Medical University of Gdańsk, Gdańsk, Poland; Praxis and Dialysis Center Zurich-City and University of Zurich, Zurich, Switzerland; Department of Nephrology, Gregorio Marañón University Hospital, Madrid, Spain; Department of Nephrology and Dialysis, Sant’Anna Hospital, ASST Lariana, Como, Italy

Cardiovascular (CV) disease remains one of the major challenges in patients with chronic kidney disease (CKD), being the leading cause of death of this population [1]. Also, in women, CV disease is the primary cause of mortality, despite their overall longer life expectancy. In general, women experience worse CV outcomes and are less likely than men to receive evidence-based therapies [2]. These gaps reflect a complex interplay of biological and systemic factors that range from greater platelet reactivity and higher rates of adverse drug reactions to later diagnosis, lower referral rates and differences in health-seeking behaviour.

**Figure 1:**
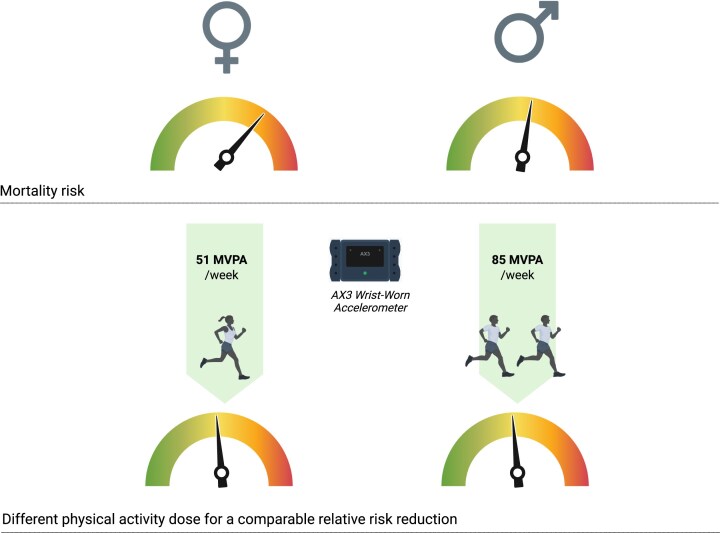
To achieve a comparable relative risk reduction in mortality (which is higher in women than in men; upper panel), men with CHD require 85 min/week of moderate-to-vigorous physical activity (MVPA) and women 51 min/week (lower panel). Based on the paper by Chen *et al*. [5].

Sex-related disparities in kidney disease are gaining increasing recognition. Women are more likely to develop CKD, progress more slowly, yet remain less frequently diagnosed, referred or treated with kidney replacement therapy [3]. In comparison with men, women face a lower prevalence of (sub)clinical CV disease in the CV–kidney–metabolic syndrome but experience excess mortality risk [4] (Fig. 1, upper panel). The biological basis of this paradox might involve differences in body composition, hormonal milieu, nitric oxide bioavailability and platelet function, which influence immune response, vascular remodelling and drug metabolism.

Reduction of sedentary behaviour and promotion of regular physical activity is crucial to improve health outcomes, particularly in high-risk populations [6]. Regular physical activity significantly reduces CV mortality in both healthy individuals and patients with established CV disease. Current cardiological international guidelines [American Heart Association (AHA), European Society of Cardiology (ESC), World Health Organization (WHO)] recommend at least 150 min week of moderate-intensity activity, or at least 75 min per week of vigorous-intensity activity. However preventive efforts remain largely insufficient, especially in women. Girls are less encouraged to participate in sports than boys, resulting in persistently lower levels of physical activity across the lifespan. The Prospective Urban Rural Epidemiology (PURE) study showed that women are approximately three times less likely than men to engage in regular exercise [7]. This holds true in different countries and is independent of income [7].

Against this background of inadequate prevention, emerging data highlight that women may derive greater CV benefit from physical activity than men. In a prospective study published in *Nature Cardiovascular Research*, Chen *et al*. investigated sex-specific associations between accelerometer-measured physical activity and the risk of incident coronary heart disease (CHD) in a CHD-free population using UK Biobank data [5]. The authors also examined the associations between physical activity and both all-cause and CV mortality among patients with established CHD. The analysis included 85 412 individuals with valid wrist-worn triaxial accelerometer data (2013–15), of whom 80 243 were CHD-free (mean age: 61.54 ± 7.84 years; 57.3% female) and 5169 had established CHD (mean age: 66.93 ± 5.90 years; 30.0% female). Among participants without baseline CHD, meeting physical activity guidelines (AHA/ESC/WHO) was associated with reduced CHD incidence in both sexes, with a significantly greater relative risk reduction in women than in men (22% vs 17%, *P*_interaction_ = .009). In those with established CHD, active females experienced greater mortality risk reduction than active males (hazard ratio = 0.30 vs 0.81; *P*_interaction_ = .004). A sex-specific dose–response pattern was observed: men required 85 min/week and women 51 min/week of moderate-to-vigorous physical activity to achieve comparable mortality risk reduction (Fig. 1, lower panel). Exceeding extended WHO recommendations (>300 min/week of moderate-intensity or >150 min/week of vigorous-intensity physical activity) did not provide additional survival benefit. Overall, the study showed that adherence to current physical activity guidelines conferred significant CV and survival benefits in both sexes, with substantially greater effects in women, regardless of baseline CHD status. The authors employed robust statistical modelling, including sex-stratified analyses with interaction testing, multiple timescales, comprehensive covariate adjustment and wide sensitivity analyses. Missing data were handled by multiple imputation, though residual confounding from unmeasured factors such as dietary habits or medication adherence cannot be entirely excluded. Mechanistically, lower baseline fitness levels in female patients and sex-specific metabolic adaptations may contribute to the observed sex differences but it is clear that more research is needed.

A key strength of the study by Chen *et al*. [5] was the objective recording of physical activity data using the validated wrist-worn AX3 accelerometer, which captures high-resolution, triaxial movement information to quantify activity intensity, duration and diurnal patterns. Compared with self-reported questionnaires, accelerometery provides superior accuracy and reproducibility, and it reduces sex-related reporting and recall bias. Nonetheless, wrist-worn accelerometers have technical limitations: they may underestimate static or low-acceleration activities (e.g. cycling or resistance training), require meticulous calibration and data cleaning to exclude non-wear periods, and could be less precise in detecting posture than thigh- or hip-mounted devices, depending on the analysis technique [8].

CKD was not included in the predefined subgroups or covariate-interaction analyses of the study by Chen *et al*. [5]. Nevertheless, hypertension was highly prevalent (49.4% of CHD-free participants and 87.5% in those with established CV disease), supporting the relevance of these findings to nephrology populations. Interestingly, analyses from the same UK Biobank showed that both device-based and self-reported physical activity measures were associated with a lower risk of CKD [9], although device-measured activity did not predict CV risk in participants with CKD (defined as estimated glomerular filtration rate <60 mL/min/1.73 m²). These associations did not differ between women and men [10]. However, the study sample was rather small, i.e. 1170 subjects with CKD.

Despite consensus on its benefits on CV health, regular exercise is still little implemented in CKD patients of both sexes. With CKD patients having unique exercise limitations and physiological needs, the availability of wearable devices for measuring physical activity reliably opens new scenarios, leading towards a more personalized approach and tailored recommendations. The future of wearable devices is rapidly evolving, with real-time and personalized recording, multi-sensor integration and artificial intelligence (AI)-driven guidance [11]. These new sophisticated devices include smart clothing, skin patches and wearable sensors capable of real-time measurement of heart rate and function, electrolyte and hydration status, metabolism and respiration [12]. The AI analysis of this huge amount of data will allow not only fitness tracking but also telemonitoring, with increased safety for vulnerable categories and real-time indications for modifying exercise intensity and pattern. In the specific context of sex differences, next-generation wearables and AI-integrated analyses could incorporate sex-specific algorithms considering differences in body size, hormonal status and CV responses, improving the accuracy of physiological measurements and risk estimation in women compared with traditional, male-derived reference models. Furthermore, the integration of cycle-aware tracking and pregnancy- or menopause-specific profiles could provide more tailored feedback on safe and effective exercise dosing for women, who have historically been underrepresented in CV research and device validation studies.

In conclusion, we have entered an era in which exercise is not just medicine: it is ‘personalized’ medicine. Sex differences must be better understood, recognized and integrated into both research and clinical practice. As a next step, sophisticated wearable devices can offer a powerful tool to support individualized exercise prescriptions while enhancing patient motivation and adherence.
